# Footprints of Middle Ages Kingdoms Are Still Visible in the Contemporary Surname Structure of Spain

**DOI:** 10.1371/journal.pone.0121472

**Published:** 2015-04-07

**Authors:** Roberto Rodríguez-Díaz, Franz Manni, María José Blanco-Villegas

**Affiliations:** 1 Área de Antropología Física, Departamento de Biología Animal, Facultad de Biología, Universidad de Salamanca, Salamanca, Spain; 2 Department Hommes, Natures, Societés, Eco-Anthropologie et Ethnobiologie, UMR 7206 CNRS, University Paris Diderot, Sorbonne Paris Cité, Musée de l’Homme, National Museum of Natural History, Paris, France; Universitat Pompeu Fabra, SPAIN

## Abstract

To assess whether the present-day geographical variability of Spanish surnames mirrors historical phenomena occurred at the times of their introduction (13^th^-16^th^ century), and to infer the possible effect of foreign immigration (about 11% of present-day) on the observed patterns of diversity, we have analyzed the frequency distribution of 33,753 unique surnames (tokens) occurring 51,419,788 times, according to the list of Spanish residents of the year 2008. Isonymy measures and surname distances have been computed for, and between, the 47 mainland Spanish provinces and compared to a numerical classification of corresponding language varieties spoken in Spain. The comparison of the two bootstrap consensus trees, representing surname and linguistic variability, suggests a similar picture; major clusters are located in the east (Aragón, Cataluña, Valencia), and in the north of the country (Asturias, Galicia, León). Remaining regions appear to be considerably homogeneous. We interpret this pattern as the long-lasting effect of the surname and linguistic normalization actively led by the Christian kingdoms of the north (Reigns of Castilla y León and Aragón) during and after the southwards reconquest (*Reconquista*) of the territories ruled by the Arabs from the 8^th^ century to the late 15^th^ century, that is when surnames became transmitted in a fixed way and when Castilian linguistic varieties became increasingly prestigious and spread out. The geography of contemporary surname and linguistic variability in Spain corresponds to the political geography at the end of the Middle-Ages. The synchronicity between surname adoption and the political and cultural effects of the *Reconquista* have permanently forged a Spanish identity that subsequent migrations, internal or external, did not deface.

## Introduction

*Llegando el autor desta grande historia a contar lo que en este capítulo cuenta*, *dice que quisiera pasarle en silencio*, *temeroso de que no había de ser creído […]*. *Finalmente*, *aunque con este miedo y recelo*, *las escribió de la misma manera que él las hizo*, *sin añadir ni quitar a la historia un átomo de la verdad*, *sin dársele nada por objeciones que podían ponerle de mentiroso; y tuvo razón*, *porque la verdad adelgaza y no quiebra*, *y siempre anda sobre la mentira*, *como el aceite sobre el agua…*

[When the author of this great history comes to relate what is set down in this chapter he says he would have preferred to pass it over in silence, fearing it would not be believed […]. But after all, though still under the same fear and apprehension, he has recorded it without adding to the story or leaving out a particle of the truth, and entirely disregarding the charges of falsehood that might be brought against him; and he was right, for the truth may run fine but will not break, and always rises above falsehood as oil above water…]

Miguel de Cervantes Saavedra, Don Quixote, second part, chapter X.

Fifty years have passed since the seminal study on the relation between surname diversity and inbreeding by Crow and Mange [1] and, today, surname studies constitute a large body of research in population genetics. The vertical transmission of surnames (generally along the male line), their availability in large numbers (telephone directories, conscription lists, voters’ list, etc.), and the large spectrum of applications they have in different disciplines (demography, genetics, geography, linguistics, history) made them a popular source of data. General background can be found in references [2, 3, 4, 5, 6, 7].

In many Europeans countries, surnames started to develop in the early Middle Ages and became stable, generation after generation, towards the 16^th^ century. Initially they were a simple way to identify a family or a person, like a designation or nickname that could vary over the time and the generations (the son of Paul, the miller, the man from the river, etc.), while later, for the administrative need to identify a lineage without ambiguities, they became progressively fixed in meaning and spelling. The geographic diversity of the naming practices, the influence of regional languages, the political asset of a region, contributed to the diversity of initial surnames that remain, several centuries after their introduction, a marker of regional diversity. Analyzing Spanish surnames as markers of population diversity, or homogeneity, is the purpose of this research.

In Spain, the use of surnames started with the 10^th^ century. They became progressively inherited from the 13^th^ to the 15^th^ century, which is during the growing expansion of the Reign of Castilla and the *Reconquista* of the territories ruled by the Arabs in the preceding centuries. Isabel I, queen of Castilla y León, and her husband Ferdinand II of Aragón (known as the Catholic Majesties) completed the reconquest and gave stability to their respective reigns in a process of large political integration that officially ended, with a unique crown, in 1714 (until this date the two reigns retained separate legal systems). At the time, religion was also a political instrument and the Spanish Inquisition (a Catholic administration existed between 1478 and 1834 and officially devoted to the preservation of religious orthodoxy) contributed to the castillanization and normalization of surnames (a process that lasted until 1870), ultimately leading to a considerable loss of surname diversity. Newly adopted surnames (often the same prototypical ones) were often ending with the suffix—*ez*, a typical Castillan patronymic form (Fernánd*ez*, Rodrígu*ez*, Gutiérr*ez*, etc.). This process corresponds to the growing of the Castilian kingdom, when Aragónese, Asturian-Leonese and Basque names were Castilianized. This is particularly the case for Aragónese and Asturian-Leonese family names. A confirmation that records were generally written in Castilian, and therefore the surnames, comes from the Spanish surnames existing in Latin America, a major destination of historical Spanish emigration, that are generally in this form. The process of Castilianization, together with the use of a double surname-system (two single-surnames A and B can lead to four double-surname combinations AB, BA, AA, BB), largely explains why Spain has a lower number of surname variants than other European countries [8, 9, 10, 11].

Castilianized forms lasted because, in the 16^th^ century, the prescriptions of the Ecumenical Council of the Roman Catholic Church (held in Trento, Italy, from 1545 to 1563) made mandatory the use of vertically transmitted surnames, stable generation after generation, in order to keep exhaustive birth and marriage records in all parishes (death and census records became compulsory in 1614).

Arab and Jewish surnames, two large communities in the Spain of that time, did not escape the “normalization” described above, meaning that a large proportion of the Arab and Jewish family names existing nowadays in Spain corresponds to later immigration, and not to the descendants of the medieval population.

By using the national telephone directory as source of data, the Spanish surname corpus has been studied at least three times [12, 13, 14], the latter study concerning a wider European frame. The clustering of Spanish regions reported in [12], according to surname similarity, was contradicted in [14] and not addressed in [13]. This is the reason why we focus on Spanish surnames again.

Our aim is to see whether the present-day corpus of Spanish surnames, that internal migrations and international immigration (Table 1) to Spain have modified, still conveys the signal of their historical origin. In this frame, Manni et al. [15] and Boattini et al. [16] have developed and validated a method to identify, from modern surname *corpora*, the family names that are historically typical (“autochthonous”) of given regions, that is those that better describe the Middle-Ages population stock in geographical terms. In this article we adopt the opposite approach by avoiding any surname selection in the analysis of the *Padrón municipal*, the largest available register of the Spanish population. The *Padrón municipal* is a national census of all residents, listed regardless of their age and nationality. It also includes all the immigrants whose administrative status has not been decided yet. This database is less conservative than telephone directories or voters’ lists that have a lower sample size and do not represent all social groups, particularly immigrants (see Table 1 and check reference [17]).

**Table 1:** Foreign population in Spain by countries of origin.

*Provenance*	*Percentage [%]*
Romania	14
United Kingdom	7
Germany	3
Italy	3
Other Europe	18
*Total Europe*	*45*
Morocco	12
Other Africa	5
*Total Africa*	*17*
Ecuador	8
Colombia	5
Bolivia	5
Argentina	3
Other Americas	13
*Total Americas*	*34*
China	2
Other Asia	2
*Total Asia*	*4*

The foreign population is 11.3% (5,220,577 individuals) over a total of 46,157,822 individuals. Source: *Padrón municipal* 2008, *Instituto Nacional de Estadistica de Espana* (INE).

Several languages are currently spoken in Spain (Table 2) and they have had an influence on surname diversity. To take this aspect into account, we report a computational analysis of linguistic features found in the linguistic atlas of the Iberian Peninsula (ALPI) [18] adapted from the original publication of Hans Goebl [19].

**Table 2:** Linguistic varieties spoken in Spain in 2014, according to the Ethnologue [35].

*Language*	*(Ethnologue code)*	*Speakers*
Aragónese*	arg	~10,000
Asturian-Leonese*	ast	~100,000
Basque*	eus	~400,000
Iberian Romani (Calò)	rmq	~40,000
Catalan*	cat	~3,750,000
Erromintxela(Basque Calò)	emx	~500
Extremaduran*	ext	~200,000
Fala	fax	~5,000
Galician*	glg	2,300,000
Gascon, Aranese*	oci	~4,000
Castilian*	spa	~38,000,000

Only the varieties followed by an asterisk are accounted in the Linguistic Atlas of the Iberian Peninsula [18] that we have reprocessed with computational linguistics methods (see Figs 4–7B).

All the computational work we present, accounting for surname and linguistic diversity, is based on pairwise distance measures between the 47 mainland Spanish provinces. The Canary and the Balearic islands have been excluded, as well as the Spanish overseas territories located at the south of the Gibraltar straight (Ceuta and Melilla).

The robustness of the surname classification has been tested by bootstrap [20]. In fact, it is our experience that regular clustering lacks stability because clustering algorithms look for the minimum distance between two points in a distance-matrix and, often, several pairs of elements show similar distances. As a consequence, small differences in the input data-matrix can lead to considerably different clusters. To overcome this instability, at least two main methods have been suggested and tested on surname and linguistic data over the years: bootstrapping [20, 21], and noisy clustering [22]. Briefly, noisy clustering can be viewed as a procedure in which different amounts of random noise are added to the distance matrix during repeated clustering, whereas bootstrapping consists of varying the input dataset in subsequent clustering iterations, allowing some surnames, or words, to be repeated. Both techniques lead to a consensus (or composite) dendrogram [23]; this is the kind of output that we will analyze. We consider the test of robustness an essential step, and it is likely that the inconsistencies between the classification of Spanish surnames noted in the papers by Rodriguez-Larralde et al. [12] and Scapoli et al. [14] might have been avoided if the authors had applied a test of this kind.

## Methods

### The surname data

**Fig 1:**
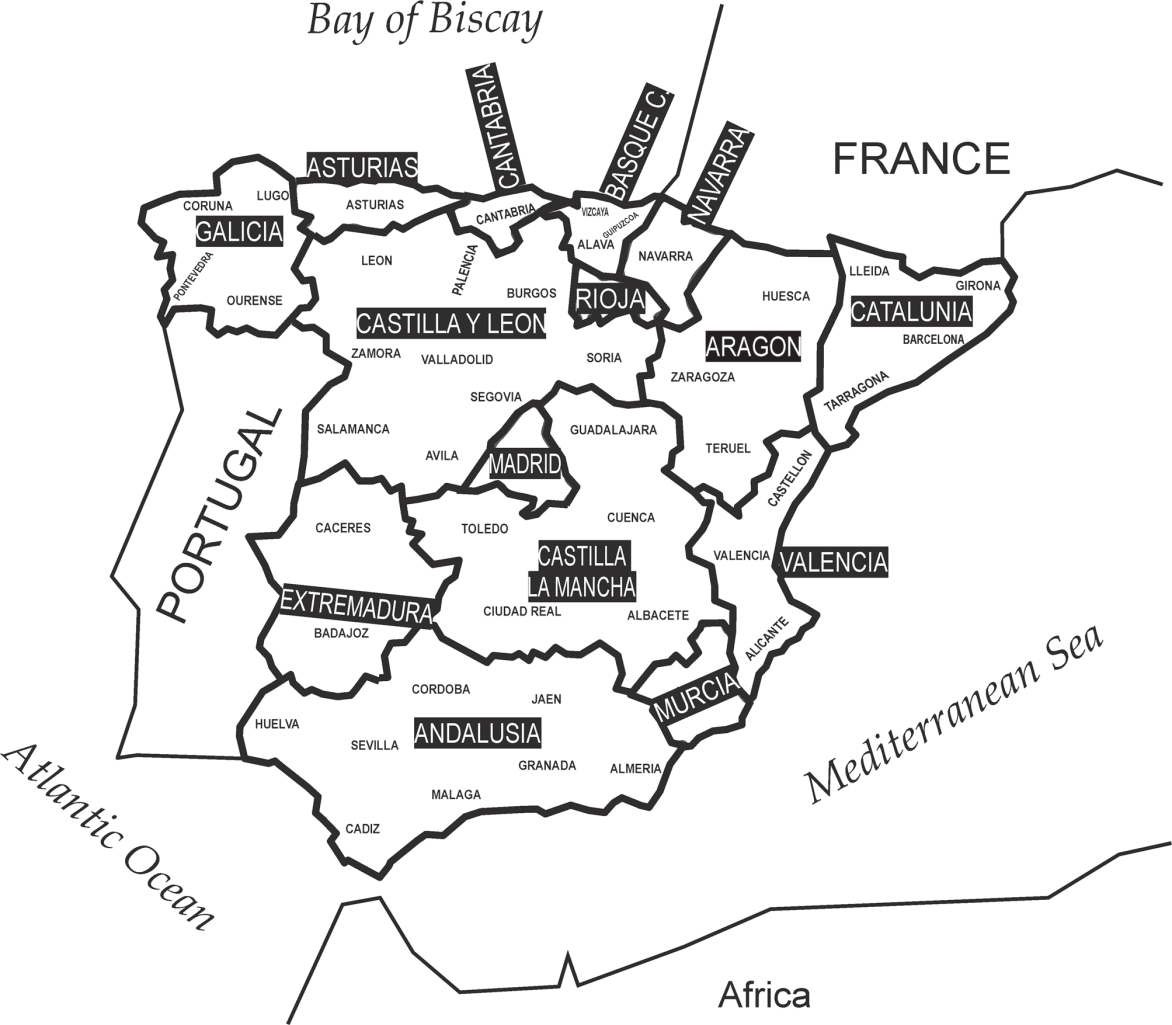
Geographic map of Spain. The names of the provinces are reported in small capital letters. The names of the regions are shown as black labels. Note that some regions consist of a single province. Spanish islands are not represented in the map because they were not studied.

The *Padrón municipal* (list of residents by municipality regardless of age and citizenship) of the year 2008 has been obtained from the Spanish National Institute of Statistics (*Instituto Nacional de Estadística*, INE—Summary statistics are freely available at www.ine.es). We processed the 45,593,365 personal records corresponding to the 47 mainland official provinces and to 15 regions (see Fig 1 and Table 3)

**Table 3:** Surname data per province and region.

***Province***	***Region***	***Municipa-lities***	***N (INE)***	***Density (ind/ km2)***	***N Tokens***	***N Tokens / N full population***	***S = Tokens***	***S/N Tokens***	***Isonymy***	***Entropy***
**Albacete**	Castilla—La Mancha	87	397493	332.53	696059	1.75	2890	0.00415	0.016301	5.612761
**Alicante/Alacant**	Valencia	141	1891477	80.16	2753076	1.46	9151	0.00332	0.007232	6.578898
**Almería**	Andalucía	102	667635	104.92	1023128	1.53	4204	0.00411	0.013206	5.69845
**Araba/Álava**	Pais Vasco/Basques	51	309635	26.95	503959	1.63	5567	0.01105	0.00724	6.705729
**Asturias**	Asturias	78	1080138	102.00	1920957	1.78	6224	0.00324	0.025725	5.427719
**Ávila**	Castilla y León	248	171815	21.45	267840	1.56	1693	0.00632	0.026546	4.974525
**Badajoz**	Extremadura	164	685246	31.90	1203833	1.76	3949	0.00328	0.007839	6.184083
**Barcelona**	Cataluña	311	5416447	715.42	301742	0.06	4061	0.01346	0.006367	6.584385
**Bizkaia**	Pais Vasco/Basques	112	1146421	520.86	1810854	1.58	9452	0.00522	0.006089	6.905668
**Burgos**	Castilla y León	371	373672	26.26	590646	1.58	3858	0.00653	0.009664	6.082197
**Cáceres**	Extremadura	221	412498	20.91	696303	1.69	2814	0.00404	0.010414	5.905415
**Cádiz**	Andalucía	44	1220467	167.21	2218350	1.82	6089	0.00275	0.007967	6.232632
**Cantabria**	Cantabria	102	582138	114.60	945248	1.62	4671	0.00494	0.012546	5.929513
**Castellón/Castelló**	Valencia	135	594915	91.13	867299	1.46	5821	0.00671	0.004314	6.8022
**Ciudad Real**	Castilla—La Mancha	102	522343	26.96	886695	1.70	3312	0.00374	0.009924	5.968138
**Córdoba**	Andalucía	75	798822	58.51	1473060	1.84	4186	0.00284	0.007301	6.120176
**Coruña (A)**	Galicia	94	1139121	145.01	2029253	1.78	4994	0.00246	0.008848	6.165282
**Cuenca**	Castilla—La Mancha	238	215274	12.79	324160	1.51	2079	0.00641	0.012363	5.844352
**Gipuzkoa**	Pais Vasco/Basques	88	701056	356.37	1066577	1.52	7283	0.00683	0.003606	7.024319
**Girona**	Cataluña	221	731864	128.04	821377	1.12	6811	0.00829	0.004763	6.82443
**Granada**	Andalucía	168	901220	73.17	1531544	1.70	3918	0.00256	0.012391	5.726686
**Guadalajara**	Castilla—La Mancha	288	237787	21.01	310916	1.31	3010	0.00968	0.011244	5.974349
**Huelva**	Andalucía	79	507915	51.52	860123	1.69	3553	0.00413	0.011199	5.771795
**Huesca**	Aragón	202	225271	14.60	276891	1.23	3530	0.01275	0.003198	6.883493
**Jaén**	Andalucía	97	667438	49.69	1206451	1.81	3193	0.00265	0.009574	5.899168
**Rioja (La)**	Rioja	174	317501	64.02	478477	1.51	4135	0.00864	0.009702	6.20085
**León**	Castilla y León	211	500200	31.95	834196	1.67	3264	0.00391	0.022666	5.34661
**Lleida**	Cataluña	231	426872	36.53	496441	1.16	5871	0.01183	0.003101	7.149126
**Lugo**	Galicia	67	355549	35.66	619753	1.74	2357	0.00380	0.022581	5.34707
**Madrid**	Madrid	179	6271638	808.32	2575022	0.41	10114	0.00393	0.009428	6.388433
**Málaga**	Andalucía	101	1563261	222.33	2508900	1.60	7438	0.00297	0.008479	6.144749
**Murcia**	Murcia	45	1426109	129.94	2426454	1.70	6650	0.00274	0.014517	5.897324
**Navarra**	Navarra	272	620377	61.03	875044	1.41	6509	0.00744	0.004311	7.012149
**Ourense**	Galicia	92	336099	45.63	581330	1.73	2147	0.00369	0.024442	5.130659
**Palencia**	Castilla y León	191	173454	21.32	272273	1.57	2006	0.00737	0.011211	5.797694
**Pontevedra**	Galicia	62	953400	214.37	1710057	1.79	5019	0.00294	0.011019	6.014782
**Salamanca**	Castilla y León	362	353404	28.58	578092	1.64	2548	0.00441	0.027121	5.137096
**Segovia**	Castilla y León	209	163899	23.72	231590	1.41	1696	0.00732	0.014007	5.549435
**Sevilla**	Andalucía	105	1875462	137.43	3385166	1.80	8302	0.00245	0.008563	6.220057
**Soria**	Castilla y León	183	94646	9.23	137558	1.45	1393	0.01013	0.010763	5.653739
**Tarragona**	Cataluña	184	788895	128.75	1020551	1.29	7542	0.00739	0.004505	6.934706
**Teruel**	Aragón	236	146324	9.77	193834	1.32	2147	0.01108	0.005673	6.233202
**Toledo**	Castilla—La Mancha	204	670203	46.01	1009269	1.51	4194	0.00416	0.014376	5.759388
**Valencia/València**	Valencia	266	2543209	240.10	2134762	0.84	7114	0.00333	0.00615	6.626234
**Valladolid**	Castilla y León	225	529019	65.95	892428	1.69	4706	0.00527	0.010633	6.084073
**Zamora**	Castilla y León	248	197221	18.31	311923	1.58	1873	0.00601	0.012807	5.631792
**Zaragoza**	Aragón	293	955323	56.34	1560327	1.63	10372	0.00665	0.003952	7.198151

[N*(INE)*] is the official population size (*Padrón municipal* 2008). [*N(tokens)*] is the number of occurrences of the [*S = tokens*] different single surnames (ex: from *Rodriguez Diaz* we obtain two tokens: *Diaz*; *Rodriguez*). Entropy is as in [36].

The INE sent us the list of surnames appearing at least five times in a single municipality, meaning that very rare surnames can be absent from the dataset.

**Fig 2:**
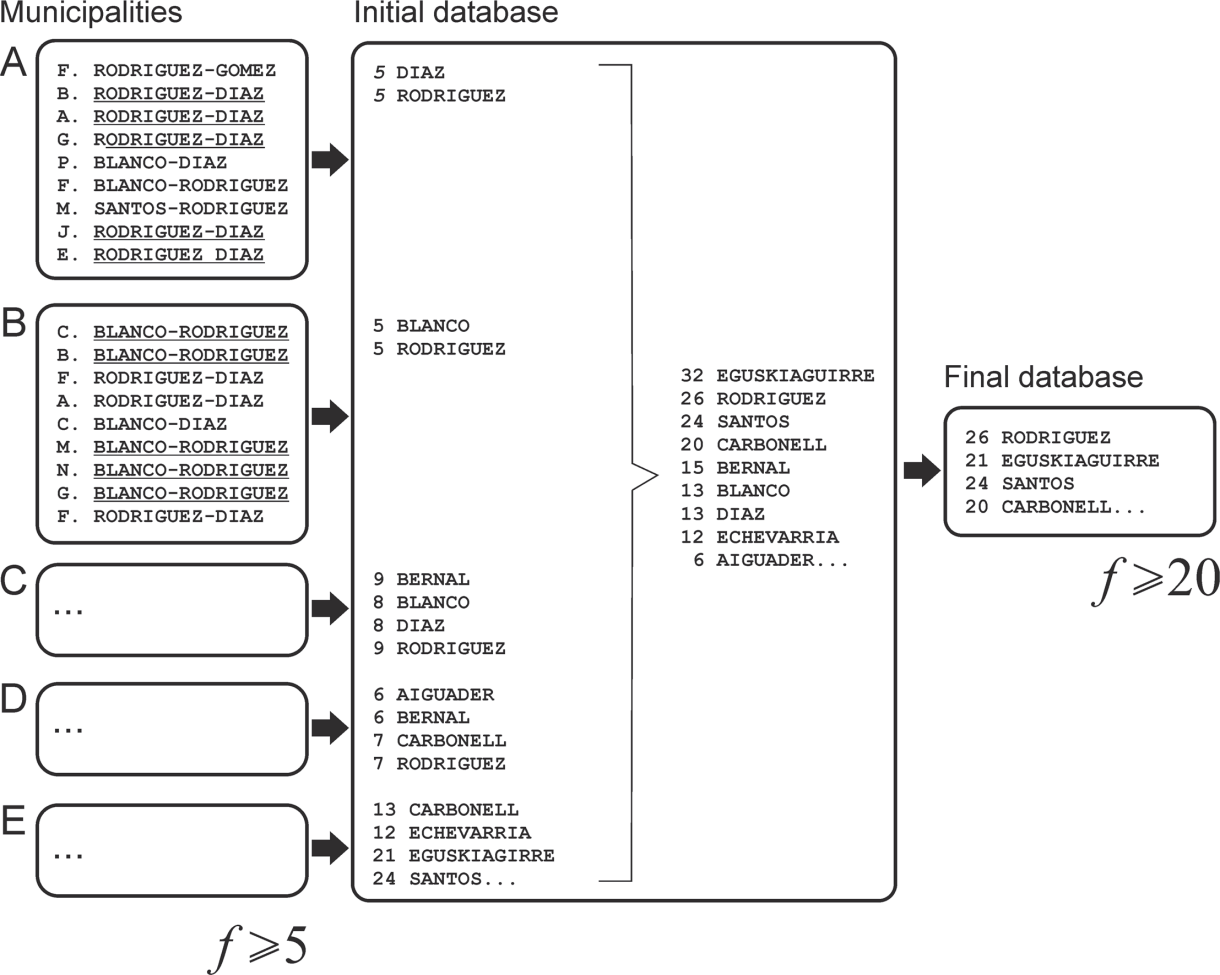
Diagram showing how surname data have been processed. Please refer to the text for a detailed explanation of the different steps.

The vast majority of Spanish surnames is double, the first one is inherited from the father (*Rodríguez*-) and the second one is inherited from the mother (-*Diaz*). At each generation the surname of the mother is lost, only the one of the father being constantly listed in the first position (recent laws now enable to swap their order or to keep only one surname of choice). Paternal and maternal surnames constantly mix in a double form that is not stable over the time and just depends on the random process of mating. This is why we decided to process them as separate tokens corresponding to single surname types (*Diaz*; *Rodríguez*) and not to individuals (*Rodríguez Diaz*). For computational ease, we kept only the surnames having a frequency higher than 20 occurrences (see Fig 2). In fact, rare surnames can be excluded because their contribution to the computation of a distance matrix is minimal.

Sometimes the records correspond to individuals having only a single surname, either because the bearers were adopted or because they immigrated from countries where a single-surname system is used. In this case, the record contributes only one surname to the tokens database (Fig 2).

The whole procedure (see Fig 2) has been the occasion to correct many obvious misspelling and digitalization errors and led to a final working database of 33,753 different single-surnames (tokens) occurring 51,419,788 times. The database is available at the following URL http://ecoanthropologie.cnrs.fr/article966.html.

### Surnames: From isonymy measures of inbreeding to surname distances

Several distance measures accounting for the diversity of two sets of surnames (pairwise difference) have been developed over the time. They are often based on isonymy, that is the probability that two surnames, randomly extracted, are identical. This probability is computed for all the different surnames in the database and depends on their frequency. In short, if two areas do not share any common surname, the measure of isonymy will be null; in the opposite extreme case, when the two areas are inhabited by individuals all having the same identical surname frequency, isonymy will be equal to 1. Mathematically isonymy is computed as:

$$I_{ij}=\sum_{s}n_{si}n_{sj}$$ (1)

where n*si* denotes the relative frequency of a given surname *s* in locations *i*, while n*sj* denotes the frequency of the same surname in location *j*. The sum is done for all the surnames.

Isonymy was originally developed to estimate inbreeding in marriage registers by computing the number of same-surname marriages, that often correspond to marriages between first-cousins. Since this application, the computation of isonymy, as a probability, has been extended to large databases.

From isonymy, several similar measures of diversity can be computed, for instance Hedrick’s [24], Nei’s [25], Lasker’s [26] and Relethford’s [27] coefficients. In this paper, at first, we have, computed the coefficients of Hedrick and Nei and, later, kept only the latter one. Hedrick’s H and Nei’s N coefficients as defined as it follows:

$$\text{Hedrick’s H =}\frac{\sum_{s}n_{si}n_{sj}}{\frac{1}{2}\left(\sum n_{si}^{2}+\sum n_{sj}^{2}\right)},$$ (2)

$$\text{Nei’s N =}\frac{\sum_{s}n_{si}n_{sj}}{{\left(\sum n_{si}^{2}\sum n_{sj}^{2}\right)}^{1/2}},$$ (3)

where parameters are defined as in (1). Distances were obtained by the following transformations:

Hedrick’s distance: d(H)=−log(H) (4)

Nei’s distance: d(N)=−log(N) (5)

We ended by having two 47 x 47 distance matrices (according to formulas 4 and 5) accounting for the 47 mainland Spanish provinces. The computations were run on the DISNEI program, written by P. Darlu. The software is available upon request to darlu@mnhn.fr.

### Test of robustness—bootstrap

Each surname has a peculiar frequency distribution in space. Any new subset of a surname database, once analyzed, leads to a distance matrix that is each time different, the differences being determined by the random presence or absence of given surnames.

**Fig 3:**
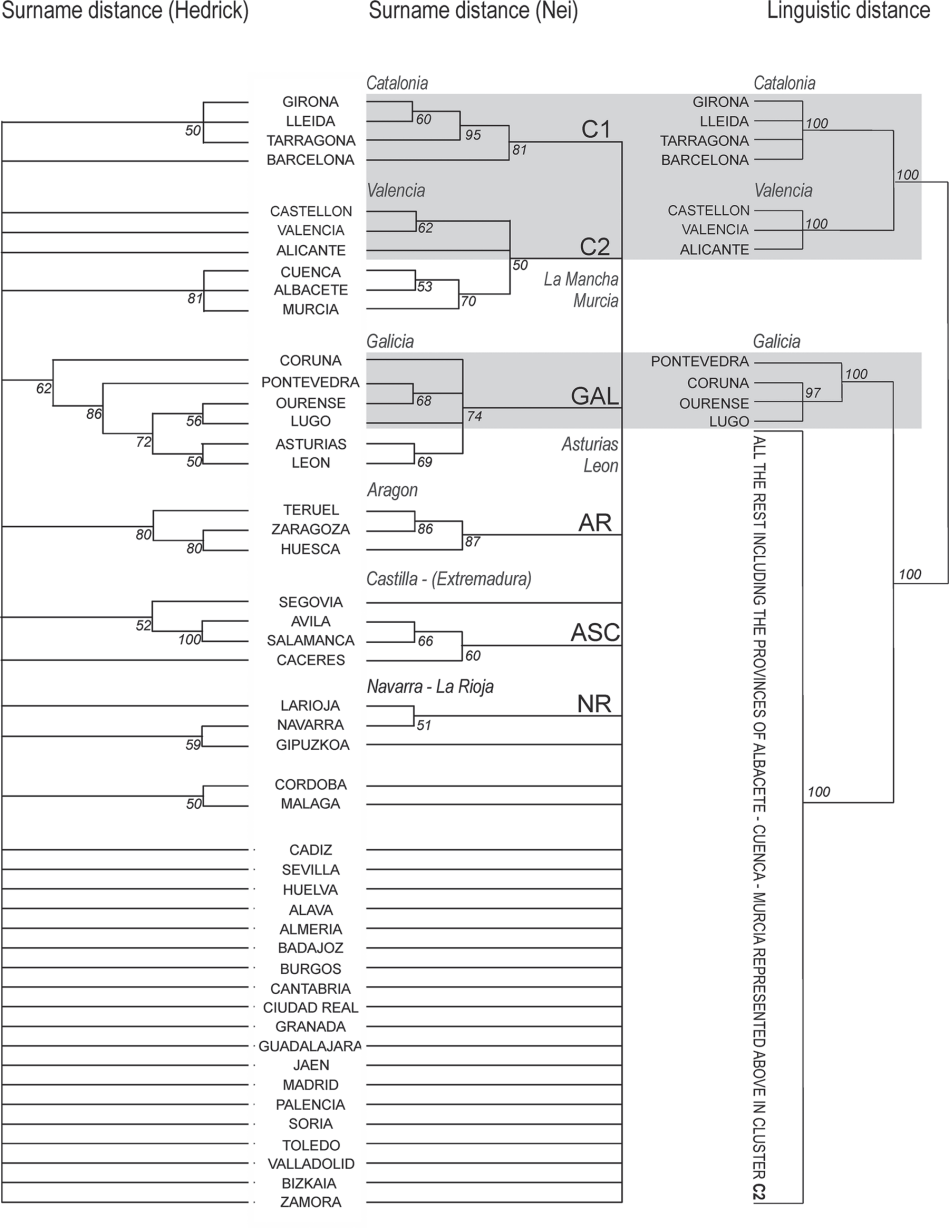
Consensus bootstrap trees [20] based on 100 Neighbor Joining [28] trees computed on the Hedrick’s [24] and Nei’s [25] surname distance. The major clusters discussed in the text (see labels) are geographically displayed in Fig 7A. A simplified linguistic tree, fully reported in Fig 4, is plot on the right. All branches having a bootstrap score lower than 50 have been collapsed.

Instead of computing a single distance matrix on the whole dataset, we preferred to resample original data to obtain 100 subsets and, from them, 100 different pairwise distance matrices. Then, from each matrix, we computed a separate Neighbor Joining (NJ) tree [28]. From the 100 trees, we computed a consensus tree where each node is scored to reflect the number of times it appears in the 100 NJ dendrograms (Fig 3). This procedure is called bootstrap and consists in resampling, with replacement, the original dataset. The essence of the bootstrap is to give different weights to the surnames in each resampled dataset. The consensus tree (Fig 3) shows how stable, through the resampled datasets, the classification is. In our case the scores, reported for each node, can vary from 1 to 100. Computations were performed by the DISNEI software and plot by Treeview [29]. Only the nodes supported by at least 50 NJ trees are shown (score ≥ 50), otherwise the branches have been collapsed (Fig 3).

**Fig 4:**
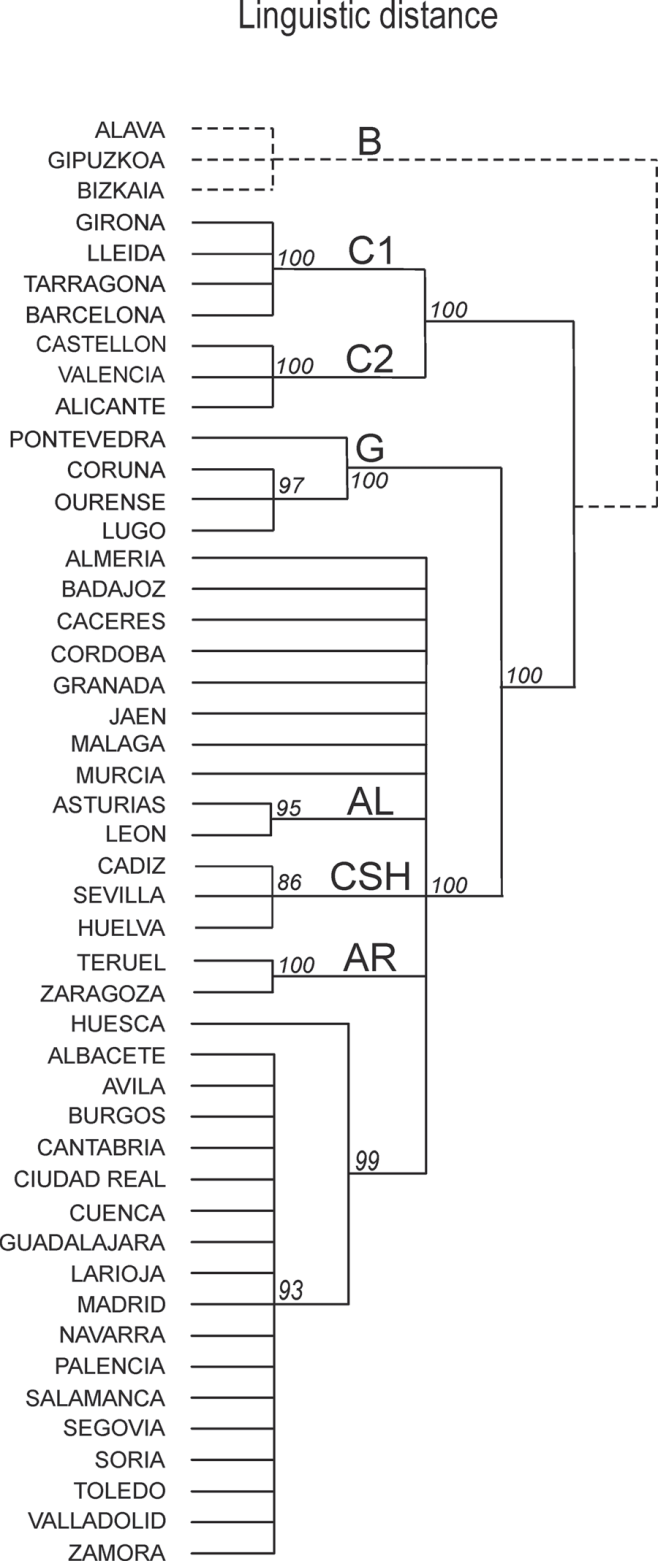
Consensus “clustering with noise” [21, 22] tree displaying Spanish linguistic varieties according to the Linguistic Atlas of the Iberian Peninsula [18] as processed in [19]. The linguistic distance (1- *RIW*) is based on the *relativer Identitätswert* [30, 31]. Major clusters (see labels) discussed in the text are geographically displayed in Fig 7B. All branches having a score lower than 90 have been collapsed. Solid lines correspond to the result of the computational analysis, while dotted lines correspond to the position that Basque varieties (absent in the ALPI) would have had after a computational classification (see text for details). A simplified version of the tree is shown in Fig 3.

### The Linguistic Atlas of the Iberian Peninsula (ALPI—*Atlas Lingüístico de la Peninsula Ibérica*)

The Linguistic Atlas of the Iberian Peninsula (ALPI) [18] is constituted by a single volume containing 75 maps accounting for 70 basilectal features in Spain and Portugal. A basilect is a variety of language, often a dialect. The atlas has not been fully published and five volumes are still missing. It is interesting to explain why.

The project was started in 1914 by the Spanish philologist Ramón Menéndez Pidal (University of Madrid, Spain) and was supervised by his student Tomás Navarro Tomás. Three teams of fieldworkers covered the Iberian Peninsula, *i*.*e*. the Catalan zone, the Castilian-speaking area and the Galician-Portuguese area. The Basque varieties have not been recorded and the islands have not been fully sampled (this is why we analyzed only the surnames of continental Spain). When the Spanish Civil War started (1936–1939) the fieldwork had almost been completed, but Navarro Tomás went in exile and took all the materials with him. The notebooks were returned to Spain in 1951, and remaining surveys were completed between 1947 and 1954. Manuel Sanchis Guarner coordinated with Lorenzo Rodríguez Castellano and Aníbal Otero the painstaking work of preparing the materials for publication, which yielded to the only available volume of the Atlas [18]. Shortly thereafter, the publishing work was suspended, and the ALPI notebooks were left (almost forgotten) in different places (private homes, different kinds of archives) until they were found and photocopied between 1999 and 2001 by David Heap (University of Western Ontario, Canada) in an epic enterprise. The remaining five volumes will hopefully be published one day, that is when all the notebooks will have been transcribed and the data cleaned. At the moment original records are available as high-resolution image files. For more details see http://westernlinguistics.ca/alpi/more_info.php

A dialectometric analysis of the first volume started in 2009 in the laboratory of Hans Goebl (University of Salzburg, Austria). Dialectometry is the subfield of dialectology aimed at mathematically measuring the differences between dialect variants. Original data have been analyzed by Goebl to identify phonetic, morphologic, syntactical and lexical features. Each feature has been processed separately in 375 working maps corresponding to 532 sampling points. Goebl sent us the final 532 x 532 similarity matrix computed according to the relative identity value (originally in German: *relativer Identitätswert*, *RIW* [30, 31]). The *RIW* measures the similarity between two basilectal varieties as the percentage of items on which the two varieties agree. This is an accepted method to measure differences between dialects and close languages. A description of the computational work is reported in [19].

### Reanalysis of the dialectometric matrix of linguistic similarity

To compare the linguistic similarity of Spain to its surname diversity, we have selected a subset of 44 sample points out of the 532 ones listed in the ALPI [18]. We kept only one linguistic variant per province (47 mainland Spanish provinces minus the 3 Basque-speaking provinces that have not been sampled in the ALPI = 44 provinces). We discarded data about Portugal. To make our choice, as many provinces are partly bilingual, we kept the variant spoken in the capital city of each province. This criterion has no special reason besides its simplicity. The selection of different sampling points might have changed our results, nevertheless a few alternative trials have shown that the main structure of linguistic diversity remains stable. We consider that the selection made, though arbitrary, is sufficient for a study addressing linguistic and surname variation at a provincial level. We stress that the selection of variants recorded in capital cities does not change a clustering that would have remained the same if other variants had been selected aside.

As we wanted to analyze linguistic diversity in terms of distance matrices, we applied the transformation:

Linguistic distance= 1 –RIW (6)

As with surnames, tiny differences in the selection of the features that are aggregated in a linguistic database can lead to unstable results (different distance matrices, different clustering). As we had no access to the dataset of linguistic features reported in [19], we could not use the bootstrap, this is why we tested the robustness of linguistic classifications with noisy clustering [21]. Bootstrap and noisy clustering give comparable results once that the level of noise, arbitrary, is set to correct values [21]. We added a random level of noise to our 44 x 44 distance matrix in order to obtain 100 “noisy” distance matrices (noise level of 0.5; 1000000 runs). As with bootstrap, we computed 100 trees and, finally, a consensus tree (Fig 4). We kept a minimal score ≥ 90 because linguistic structures are generally more robust than surname ones. To the consensus tree reported in Fig 4, we have added the Basque provinces excluded in the ALPI, according to the position they would certainly have had after a computational analysis. This artifice is fully justified because the Basque is one of the most divergent languages of all Europe and certainly the most divergent of the whole Iberian Peninsula, meaning that any computational method would classify the Basques as an outgroup.

## Results

### Surname diversity in Spain—General statistics

The working database was constituted by 33,753 tokens (single surnames) occurring 51,419,788 times. This quantity exceeds the number of individuals constituting the Spanish population in 2008 (46,157,822 according to the *Padrón municipal*) because we added the paternal and the maternal surnames to the database separately (Fig 2). If we had considered the full population, and if each resident had a typical Spanish double surname, we would have had more than 92 million occurrences (46,157,822 * 2 = 92,315,644). The theoretical discrepancy existing with the working database we processed is of ~ 41 million missing occurrences; one explanation for it is that many foreigners do not have a double surname (unless coming from Latin American countries using a Spanish double-surname system—see Table 1). In 2008, foreign residents officially were 11.3% of the total, and 66% of them came from countries using a single-surname system, meaning that they can account for ~ 3.5 million missing occurrences (Spanish citizens that do not have a double surname, either because adopted or by deliberate personal choice are a small minority). The choice of the Spanish national institute of statistics (INE) to provide only data corresponding to individuals having a surname appearing at least 5 times within each municipality (to preserve anonymity), and our decision to analyze only the surnames occurring 20 times (see Fig 2), account for the remaining larger fraction of the discrepancy (~ 37 million). This said, and while our sample remains largely representative of the Spanish population (we processed ~58% of the maximal expected number of occurrences), we noticed that the aggregation of municipal data, at the provincial level, leads to a lower sample-representativeness for some provinces (see ratio N _*tokens*_ / N _*full population*_ in Table 3).

**Fig 5:**
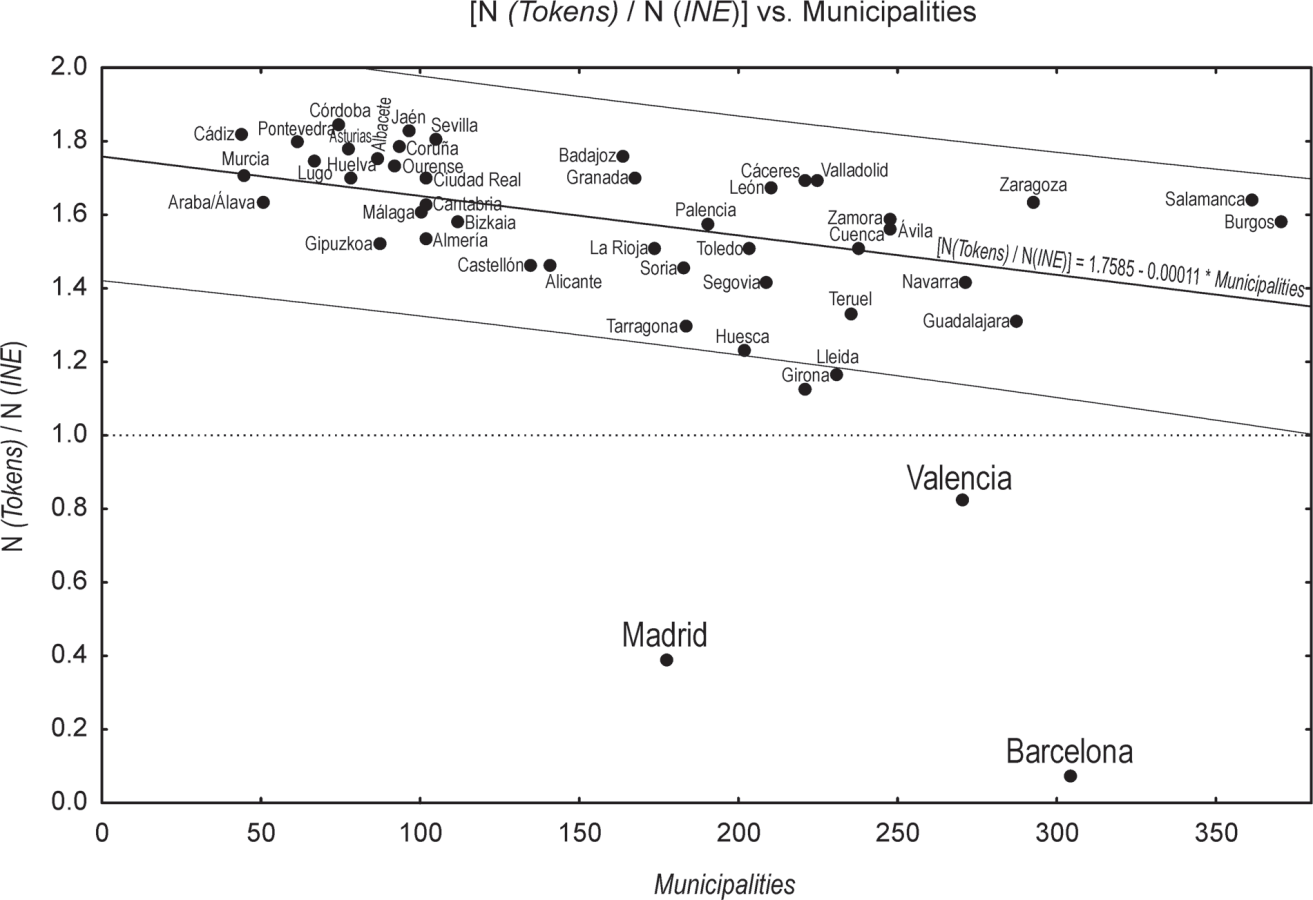
Linear regression between the ratio *N(tokens)/N(full population)* against the total number of municipalities per province (Table 3). The ratio *N(tokens)/N(full population)* is reported in Table 3 and corresponds to the number of single surnames, the tokens of the final database (see Fig 2), divided by the total number of individuals listed in the full data source (*Padrón municipal* of the year 2008 released by INE, the Spanish national institute of statistics). The trend suggests that the sample-representativeness decreases when the number of municipalities in each province rises, becoming very low for the provinces of Barcelona, Madrid and Valencia. See text for details. The regression has been computed by excluding the latter provinces.

In general, the N _*tokens*_ / N _*full population*_ ratio is above 1, meaning that our provincial samples correspond to at least 50% of the population (the nominator is theoretically expected to be twice the denominator because of the double-surname system). Very large deviations from this reasonable pattern concern the provinces of Barcelona and Madrid, and, to a lesser extent, the province of Valencia (Fig 5). These three provinces are those hosting the three biggest agglomerations of Spain. As the proportion of foreign residents bearing a single-surname type is higher in large cities because they generally attract many foreign migrants (~20% in Barcelona and Madrid), the INE cut-off of surnames with an absolute frequency lower than 5 bearers leads to a bigger loss of data in large municipalities (like the city of Barcelona consisting of a single municipality of about 1,6 million of residents) than in small ones (like Bellprat, province of Barcelona, ~ 100 inhabitants).

If the size of a municipality leads to a bias, the number of aggregated municipalities constituting each sample per-province leads to another. In our database, the provinces consisting of a large number of municipalities are less representative of their actual population-size than those consisting in a low number of municipalities. The ratio N _*tokens*_ / N _*full population*_ (Table 3) shows a highly significant linear inverse correlation with the number of municipalities (R^2^ = 0.23; p = 0.000544; see Fig 5). To test whether the increased level of missing surnames, at the municipal level, has an influence on the general classification (Fig 3), we have experimented by processing only highly frequent surnames (*f* > 60; *f* > 80; *f* > 100). It turns out that the measures of isonymy, and the clustering derived from them, remain very similar to the ones presented in this paper (*f* ≥ 20). This is not surprising, because the mathematical definition of isonymy (and related distance measures) gives a low weight to infrequent surnames. While there is a systematic bias linked to the number of municipalities that compose each province, it does not seem to significantly influence our results because, after all, the classification of Valencia, Madrid and Barcelona (Fig 3) makes sense both historically and geographically. This said, the low sample size concerning Barcelona is probably related to other biases that we could not identify, and a larger sample size might have improved its clustering.

### Isonymy levels

**Fig 6:**
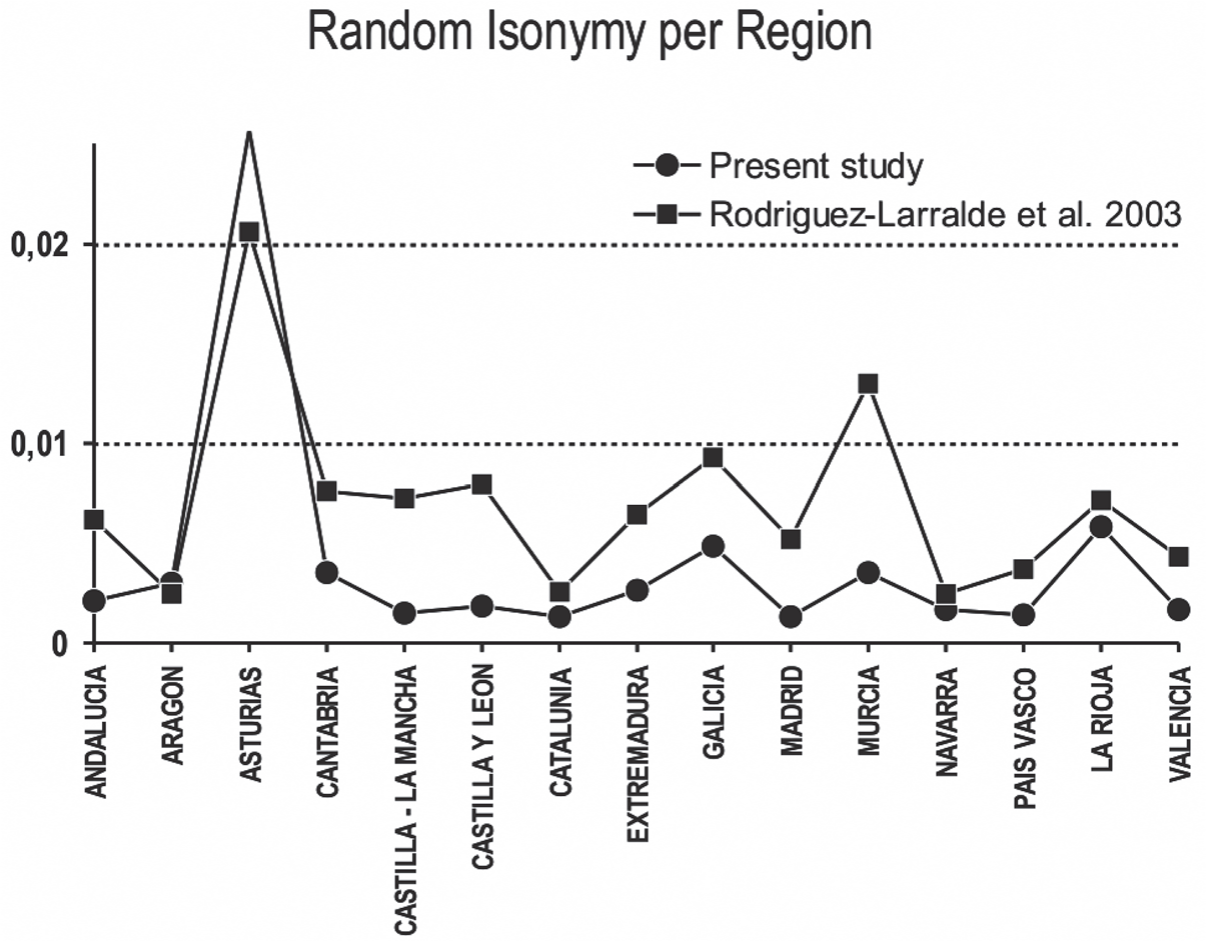
Isonymy levels per region. Please see Table 3 and Fig 7C for details and refer to isonymy values reported in [12]. Regions are listed in alphabetical order.

**Fig 7:**
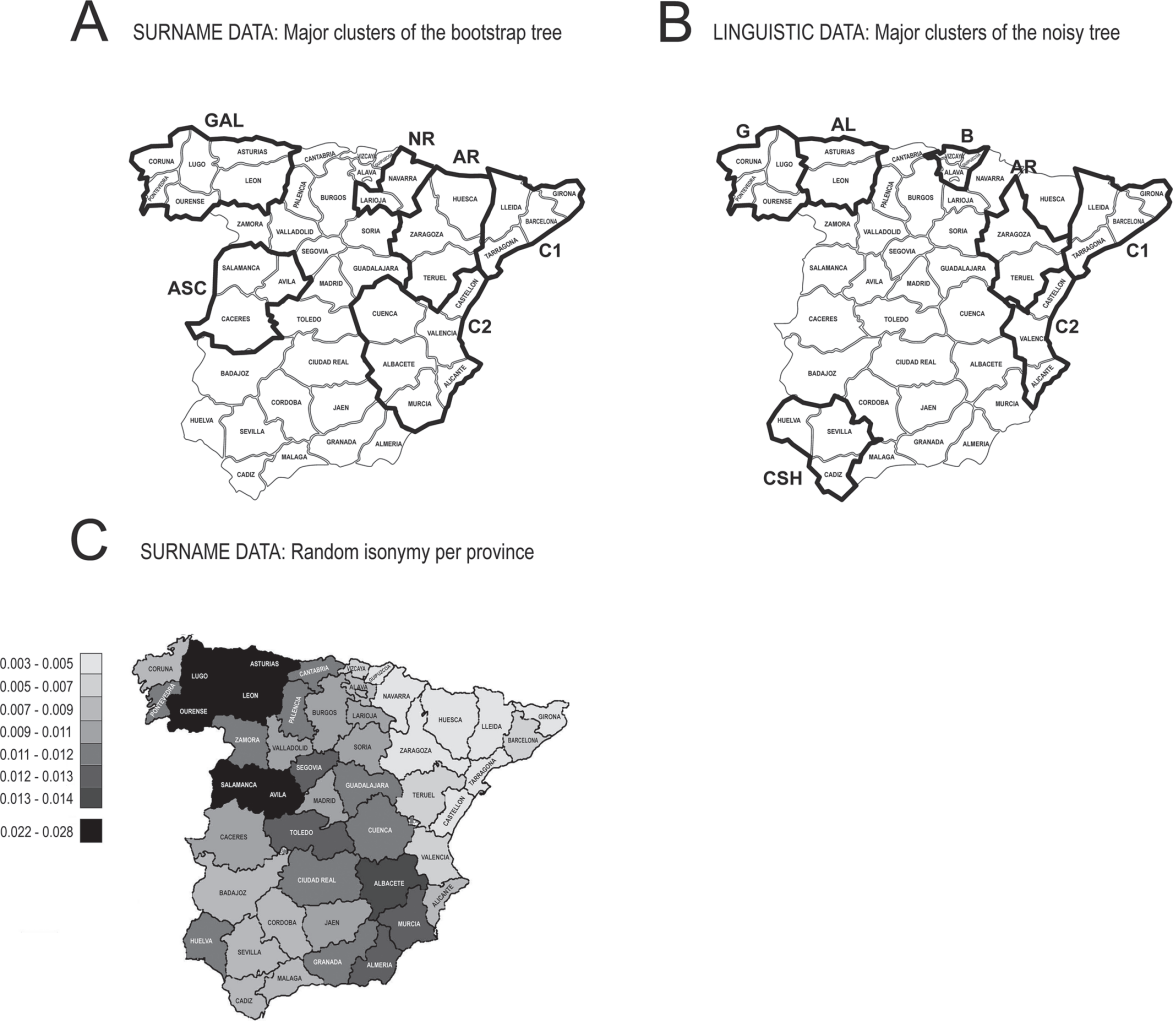
Geographical plot of the major clusters appearing in the surname (A) and linguistic (B) consensus trees of Figs **3**–4 (Nei’s) and labeled accordingly. In (C) we have plotted isonymy values (see Table 3) according to a 8-class interval; the latter class (solid black) represents an interval that is not continuous with the preceding one. Note that a same label in one map may not correspond to the same provinces in the other, see dendrograms for details.

Concerning random isonymy (Table 3; Figs 6–7C), we noticed an interesting geographic pattern with low and quite homogeneous levels in the eastern part of Spain (regions of Aragón, Cataluña, Valencia) and higher values in the rest of country. Isonymy is quite high in whole Castilla (regions of Castilla—La Mancha and Castilla y León) and in the provinces of Ávila, Asturias, León and Salamanca. Galicia also exhibits very high levels of isonymy. To be sure, there is a strong linear inverse correlation between isonymy and entropy measures (mathematical definition given in the caption of Table 3) accounting for the diversity of surnames. It means that the provinces with the highest random isonymy generally have a lower number of surnames, though this cannot be directly inferred by the average number of surnames per person (S/N in Table 3).

**Fig 8:**
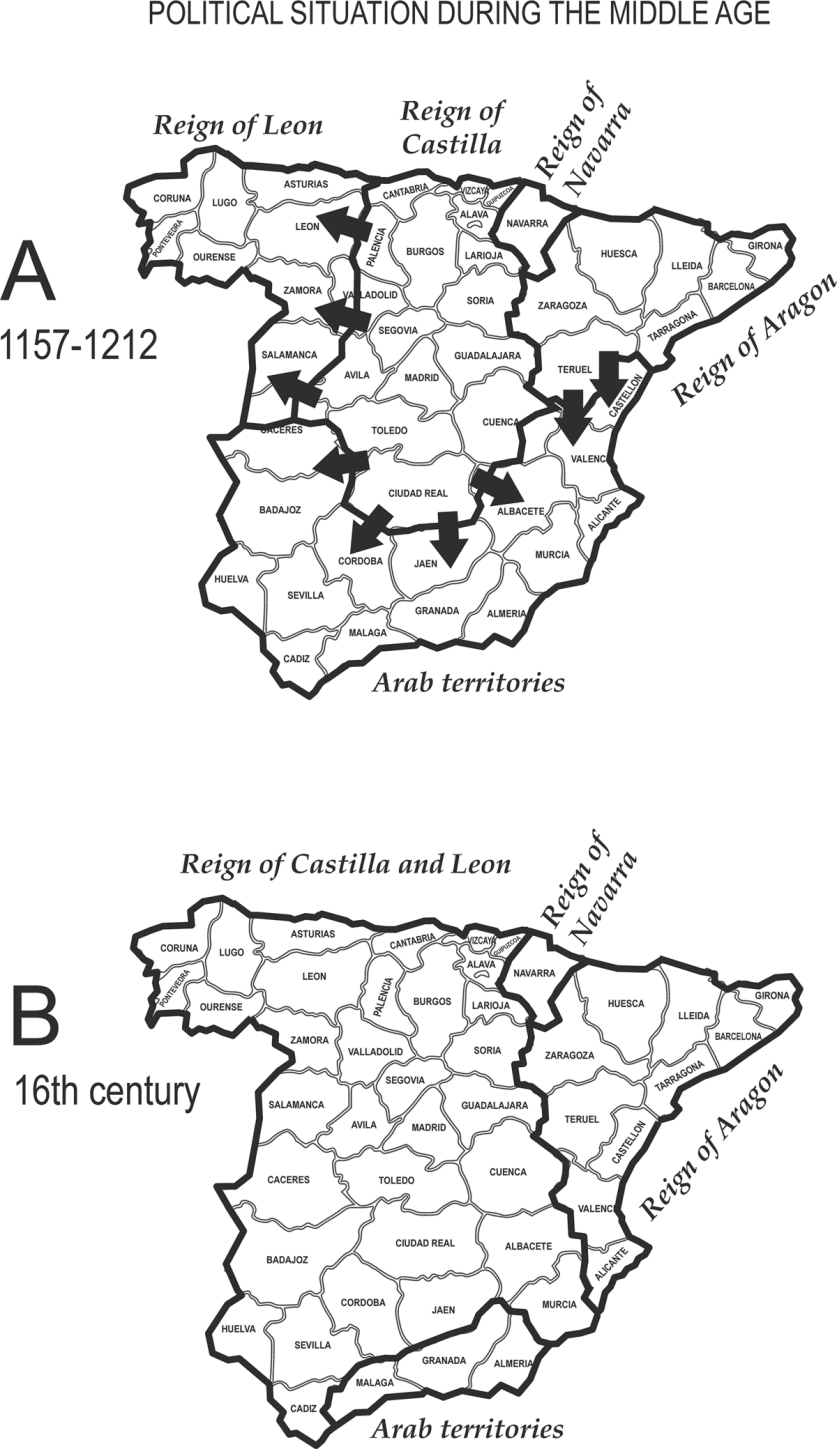
Political subdivision of Spain during the early and late Middle-Ages. In (A) is reported the political geography of the second half of the 12nd century, (B) corresponds to 1492 CE. The timeframe matches to the origin of Spanish surnames. By comparing the two maps, the expansion of the reigns of Castilla and Aragón towards the southern territories ruled by Arabs is well visible (see arrows). This process is known as the reconquest (*Reconquista*). Please note that the reigns of Leon and Castilla, independent in (A), later merged (B). The reign of Navarra remained unchanged.

While a rural lifestyle and geographic isolation can partially account for this general pattern in the provinces of Asturias, León and Salamanca, we suggest that the contrast between eastern and western provinces might, also, rely on a structural phenomenon concerning all Castilla, but not the Catalan zone, as these where two reigns (Fig 8) that kept administrative independence for a long time after their political integration (post 1492 CE). We will come back on this aspect in the discussion section.

### Surname diversity in Spain—clustering

From isonymy we computed Hedrick’s [24] and Nei’s [25] pairwise distances between all the 47 mainland Spanish provinces and, finally, a bootstrap consensus tree corresponding to them (Fig 3). While the structure of the two trees is similar, the Hedrick’s classification yields a smaller Catalonian cluster (cluster C1) and does not support the existence of a unique cluster for the region of Valencia, (differently from the Nei’s classification, see cluster C2—Alicante, Castellon, Valencia). Further, while the group Albacete-Cuenca-Murcia is highly supported in both trees, its clustering with the region of Valencia is not well supported in the Hedrick’s tree (bootstrap score below the cut-off of 50% (Fig 3). Another inconsistency between the trees concerns the clustering of Navarra, that the Hedrick’s method puts together with the Basque province of Guipuzcoa, while the Nei’s method groups with the province of La Rioja (see cluster NR in Fig 3). A last difference concerns the ASC cluster (Fig 3), where Ávila and Salamanca (represented together in both trees) are put with Segovia with the Hedrick’s approach and with Cáceres with the Nei’s classification. About the similarities of the trees, Albacete, Cuenca and Murcia are always clustered together. Further, the provinces of Galicia (GAL) and Aragón (AR) form coherent clusters. Whatever the distance adopted, we note that no specific Basque cluster appears when bootstrap scores are set to be ≥ 50. From now on, we will discuss only the Nei’s distance consensus tree because it represents larger clusters that the Hedrick’s one. In the end, and besides some change in the NR and ASC clusters (Fig 3), the Hedrick’s tree does not contradict the Nei’s representation, it just provides less support to it.

To summarize, the geographic areas corresponding to coherent surname clusters concern only a small part of continental Spain (see Fig 7A), that is Cataluña, the region of Valencia, Aragón, Galicia, Asturias-León and La Mancha-Murcia. We remind that La Mancha is a geographical and historical region that currently does not have any administrative status (it falls in the macroregion called Castilla-La Mancha). La Mancha was formed by portions of the the provinces of Albacete, Cuenca, Ciudad Real and Toledo. When we have referred to La Mancha we meant only its eastern part, that is Cuenca and Albacete.

Interestingly, no clusters with a bootstrap score higher than 50 are found in a very large area concerning central and southern Spain (Fig 7A).

### Linguistic diversity in Spain

The computational classification of Spanish linguistic varieties is accounted by the consensus tree displayed in Fig 4. We remind that the Linguistic Atlas of the Iberian Peninsula (ALPI) [18] contains no data concerning the three Basque-speaking provinces of Alava, Gipuzkoa and Bizkaya. They are reported in the tree according to the expected position they would have had if data were available (dotted branches—see the methodological section of the paper). The first partition of the tree concerns the Catalan speaking area. This cluster is divided in two subgroups (C1 and C2 in Fig 4), respectively corresponding to Cataluña and to the region of Valencia. Another very robust cluster concerns the four provinces of Galicia (G in Fig 4). All remaining provinces are put together in a very large cluster, quite unstructured. Within it, Asturias and León are together (AL). The same happens concerning three southern provinces (CSH—Cádiz, Sevilla, Huelva) and a part of the region of Aragón (provinces of Teruel and Zaragoza, but not Huesca).

Similarly to the surname classification, there are no robust clusters in a large part of continental Spain. The larger fraction of the linguistic diversity is located in the north and in the east (Aragónese, Asturian, Basque, Catalan and Galician languages). Further analyses (not shown), restricted to the Castilian speaking area only, have confirmed its really low differentiation.

The linguistic tree (Fig 4) is extremely robust and its coherence with the surname classification is apparent (Fig 7A-7B).

### Mantel correlations

When distance (or similarity) matrices concerning the same elements are available, is it common practice to compute Mantel test correlations [32]. We compared geographic, linguistic and surname distance matrices and the results are reported in Table 4.

**Table 4:** Mantel correlations between geographic, surname (Nei’s distance) [25] and linguistic distances (1-RIW] [30, 31].

***[Distance]***	*Geographic (linear)*	*Geographic (road)*	*Surname (Nei’s)*	*Linguistic (1-RIW)*
*Geographic (linear)*	1	0.986 **	0.281 *	0.598 **
*Geographic (road)*	0.986 **	1	0.277 *	0.599 **
*Surname (Nei’s d*.*)*	0.281 *	0.277 *	1	**Not significant**
*Linguistic (1-RIW)*	0.598 **	0.599 **	**Not significant**	1

Significance levels, computed according to [38], are reported as asterisks:

(*) = 0.01;

(**) = 0.001.

According to the Mantel test, while linguistic and surname measures are correlated with geographic distances, they are not cross correlated (Table 4). This result contrasts with the similar clustering they show in Fig 7. To explain the incoherence, we remind that sometimes pair-wise distances account for noise, like in the Castilian area (not elsewhere) where historical phenomena linked to the *Reconquista* have defaced a large part of its surname and linguistic variability. Our point, here, is to show that Mantel correlations are often insufficient to describe a phenomenon that concerns only a part of the pair-wise elements.

## Discussion

### Variability of Spanish surnames: Patterns of diversity

Our initial research question was to assess whether the present-day geographical variability of Spanish surnames mirrors historical phenomena occurred at the times of surname introduction (13^th^–16^th^ century), or if internal migration and international immigration have defaced them. Our analyses may be unrepresentative of the surnames (often rare) corresponding to international immigration, because we processed only a subset of the *Padron Municipal* (see Fig 2). To estimate the proportion of non-Spanish surnames, as an automatic classification is not possible, we proceeded empirically, by asking two Spanish colleagues to retrieve them in a printed version of the database. It appears that the percentage of surnames that are not Castillan/Catalan/Basque/Galician is ~ 7% of the total, in good agreement with the official count of the foreign population in 2008 (~11% in Table 1). The discrepancy of about 4% between our estimate and the official one, is easily accounted by the proportion of immigrants from Latin America (34% of the total, see Table 1), that often have a typical Spanish surname and escape detection. The conclusion is that the database is representative of the contemporary population of Spanish residents, immigrants included. This conclusion, counterintuitive given the frequency cut-off we applied to the initial data (see Fig 2), makes sense given the nature of the *Padrón Municipal*. Its records correspond to individuals of all ages; meaning that the surname of a family of immigrants constituted by three generations (grandparents, husband and wife, children) appears several times in the *Padrón Municipal*, thus making possible their inclusion in our working database.

Notwithstanding a large immigration, the surname structure of Spain largely reflects the political asset at the times of surname adoption, at the end of the Middle-Ages. In fact, the borders of the Reign of Aragón almost exactly correspond to three major clusters of surnames and the same can be said for the Reign of Navarra (respectively C1/C2/AR and NR in Fig 7A—see also Fig 8). The former Reign of León well corresponds to the surname clusters GAL and ASC in Fig 7A.

*Does this mean that the Spanish population remained largely unchanged over the centuries*? According to its surname structure it did, but this is also the effect of the surname normalization posterior to the *Reconquista*, together with the increased prestige of the Castilian language and identity. Actually these aspects hide a more mixed genetic background of the population. For example, the genetic signature of Sephardic Jews (expelled in 1492 CE), North African Muslims and other groups largely present in Spain until the reconquest, is still found in the Spanish population, as a comprehensive Y-chromosome study has recently shown [33]. If specific haplogroups are recognizable, their frequency varies geographically, being lower in Cataluña and along the corridor of the Aragónese expansion southwards. Though large, the genetic sampling of Adams et al. [33], conducted by region, does not overlap well with our surname sampling consisting in the aggregation of municipal data by province. We believe that a deeper comparison between the diversity of the Y-chromosome and the diversity of surnames would be most appropriate, given that the two markers share a similar inheritance along the male line.

Even if the historical signal is well preserved in contemporary Spanish surname data, some features of the classification (Fig 3) are likely to be of recent origin, like the absence of a Basque surname cluster. Actually, it has been shown that Basque surnames, contrary to the Catalan and Galician ones which are scattered over an area corresponding to their respective languages, are distributed in a region larger than the Basque-speaking domain. Besides the provinces of Alava, Gipuzkoa and Bizkaya (Basque linguistic core-area in Spain), Basque surnames are found in large numbers also in Aragón, Cantabria, Castilla y León and La Rioja [13]. As the Basque has never been spoken in the latter provinces [34], it is likely that many families migrated outside this speaking area after the Middle-Ages and, probably, quite recently. In addition to this dispersal, the Basque country, a long established industrial area, has attracted a large number of immigrants from other Spanish provinces and from foreign countries. The balance of the two phenomena prevents the existence of a robust Basque surname cluster.

### Variability of Spanish surnames: Patterns of isonymy

Isonymy levels at the provincial level (Table 3; Fig 7C), are lower in the eastern part of the Iberian Peninsula (Cataluña, Valencia, Aragón and Navarra) and generally higher elsewhere, with extremely high values in Galicia, Asturias, León, Salamanca and Ávila. High values of isonymy are in agreement with the loss of surname diversity in the Reign of Castilla after the *Reconquista*, as the consequence of a long-lasting process of Christianization and castillanization together with the increase of the Castilian prestige. The other way round, the lower levels of isonymy in Cataluña, Valencia, Aragón and Navarra can be interpreted by a lower degree of surname Castilianization (these regions were part of the reign of Aragón that maintained separate administrative systems until 1714 CE) and by the specific language context (corresponding to the Aragónese and Catalan language) that acted as a source of surname diversity. To these structural aspects, we should add the effect of internal migrations, from all Spain, to the wealthy province of Valencia and to the region of Cataluña (but also to Madrid and the Basque country), with a recent increase in their surname diversity. The reverse probably happened in provinces having very high levels of isonymy (Lugo, Pontevedra, Asturias, León, Salamanca and Ávila). Interestingly, and as a partial explanation deserving more research, none of the provinces listed has, or had, a special economical attractiveness. We note that isonymy also depends on the population size at the time of surnames introduction, and it is likely that high levels of isonymy reflect a sparse population during the Middle-Ages (Galicia, Asturias, León, Salamanca and Ávila).

As a general conclusion, isonymy cannot be readily attributed to a single cause and multidisciplinary research, involving historians and historical demographers, would be of help to assess the magnitude of each single factor. What is clear is that the east/west divide in the pattern of isonymy (Fig 7C) perfectly corresponds to the historical border separating the reign of Castilla y León from the reign of Aragón (Fig 8). We believe that the levels of isonymy in the eastern part cannot be directly compared to those in the western part, because they correspond to political and administrative differences that distorted the demographic processes they are expected to readily mirror. This is why we suggest that the low isonymy computed in Cataluña, Aragón and Valencia may not correspond, in reality, to a lower level of consanguinity than the western Castilian area. In Spain, the estimation of the actual levels of consanguinity from isonymy requires great caution.

We found no statistical support for the inverse correlation between consanguinity values (computed from isonymy) and the density of the population per province (Table 3) reported in [12]. To be sure, we have checked our isonymy measures against those in [12] and, despite a difference in the data source and geographic sampling (these authors did not compute isonymy per province but only per city and region), we found a substantial agreement (Fig 6). However, we note that the clustering in [12] is very different from the one we have obtained (Fig 3), and we could not replicate, at all, their dendrogram classification, even when using the distances and clustering algorithms they applied. Interestingly, the same authors [14] later reprocessed their surname data (telephone directory) and provided a second clustering that is different from the first one. As both published trees [12, 14] have not been tested for their robustness, for example by bootstrap or jackknife methods, they might not portray a solid trend in the data. This is the reason why we will not further comment on them.

### Linguistic diversity

The data contained in the Linguistic Atlas of the Iberian Peninsula (ALPI) [18] are about eighty years old, meaning that they concern linguistic varieties that have meanwhile changed. With respect to the ALPI, the phenomenon is also linked to the political regime guided by General Francisco Franco from 1939 to 1975. Franco pursued strong nationalistic policies that weakened regional identities. His regime discouraged the use of regional languages and Castilian (Spanish) was the only official and accepted way of expression. The advent of democracy, about forty years ago, has corresponded to a strong will to embrace regional cultural identities and to obtain some political and economical independence from the central government, essentially in the Basque region and in Cataluña. Some regional languages (Basque, Catalan, Galician) are now officially accepted, used at all the levels of the public life, including the medias, and taught at school. As a result, they are converging towards a norm.

The clusters yielded by the computational linguistics analysis of the ALPI (Fig 4) largely correspond to the known regional varieties of Cataluña, Aragón, Galicia, Asturias and León. The novel aspect concerns the large homogeneity of Castilian language varieties, a homogeneity that cannot be attributed the any modern leveling because in the 1930s (when the ALPI sampling was carried out) the Spanish lifestyle was still quite traditional and rural. Therefore, the reason for the low level of variation between a majority of the Castilian varieties must be older.

Before the Arab invasion, the linguistic landscape of Spain consisted of local varieties resulting from the adoption of Latin by populations that previously spoke Celtic and Iberian languages. Latin varieties evolved for about a thousand years, from the Roman conquest of the Iberian peninsula (started in 218 BCE) to the Arab take-over (started in 711 CE), when a progressive linguistic arabization started.

The length of the Arab domination, and its influence on the language, differ geographically, having lasted a couple of centuries in the North of Spain and about eight centuries in the very South. During the progressive reconquest of the peninsula by the Christian kingdoms of the North and the growing expansion of the reigns of Castilla y León and Aragón (Fig 8), northern Castilian and Catalan varieties spread to the South, thus replacing a large part of the linguistic varieties encountered. This is why the differences found in the Castilian-speaking area are known to be secondary, in other words occurred in a more recent and shorter time than the first process of differentiation from Latin [35]. The political prestige of the Castilian crown, together with the religious and cultural “normalization”, kept Castilian quite homogeneous and led, at the same time and as we mentioned already, to the large Castilianization of surnames, that are a specific part of language. The homogeneity of Castilian underlines the effectiveness of the political power of monarchies that have been able to vigorously keep a Castilian linguistic norm that has remained a key element of the identity of this nation until the end of the regime of General Franco.

By looking at the forces here at play in the forge of a distinctive Spanish linguistic identity, we can understand how strong must have been the Castilianization of Spanish surnames.
